# Isotopic heterogeneity in U.S. Urban water supply systems reflects climatic, environmental, and sociodemographic factors: Implications for forensic identification

**DOI:** 10.1371/journal.pone.0311741

**Published:** 2024-11-11

**Authors:** Chris Stantis, Alejandro Serna, Kirsten Verostick, Brett Tipple, Anne Jefferson, Gabriel J. Bowen

**Affiliations:** 1 School of Anthropology, Political Science and Sociology, Southern Illinois University, Carbondale, Illinois, United States of America; 2 Department of Geology and Geophysics, University of Utah, Salt Lake City, Utah, United States of America; 3 Department of Archaeology, BioArCh, University of York, York, North Yorkshire, United Kingdom; 4 Facultad de Ciencias Naturales y Museo, División Arqueología, Universidad Nacional de La Plata, La Plata, Buenos Aires, Argentina; 5 FloraTrace, Inc., Salt Lake City, Utah, United States of America; 6 Department of Earth Sciences, Kent State University, Kent, Ohio, United States of America; 7 Rubenstein School of Environment and Natural Resources, University of Vermont, Burlington, Vermont, United States of America; Goethe University Frankfurt: Goethe-Universitat Frankfurt am Main, GERMANY

## Abstract

The forensic application of stable oxygen isotope data from human tissues depends on naturally occurring isotopic variation in drinking water across geographic areas. One factor which complicates interpretation of forensic data is local variability: if a wide range of drinking water values is in a small geographic region it may be difficult to identify or rule out that region as a location of origin. We examine data from community collection programs documenting tap water isotope variation within 30 cities\developed areas throughout the United States. Isotopic variation within individual developed areas ranged widely, from essentially nil to greater than 9‰ (δ^18^O interdecile range). Many (14/30) of the study areas exhibited multi-modal isotope distributions, even in cases where the isotopic range was very small (e.g., <2.0‰), suggesting that the use of multiple, isotopically different sources was common. Most variation was attributed to differences in the source-region altitude or degree of evaporation for different water sources, and we observe limited variation in areas where contrasts in these variables are small. Variation tended to be largest in cities across the western USA. We explored correlations between the magnitude of local isotopic variation and climatic, environmental, and socioeconomic variables. We found that higher levels of variation occurred at sites where local water resources were likely to be more isotopically heterogeneous and in lower supply, consistent with the development of supply systems in these areas that access diverse and isotopically heterogeneous water resources. We also found that variation was positively correlated with larger developed areas, suggesting that pressures related to city growth may affect the degree to which infrastructure tapping diverse water resources is developed. Collectively, our results suggest that the influence of local tap water isotope heterogeneity on the precision of forensic geolocation is systematic and somewhat predictable, information to be factored into future applications.

## Introduction

The reconstruction of mobility histories and/or the provenance of unidentified human remains using oxygen stable isotope values (δ^18^O) of body tissues is now an established technique in forensic research [1–5]. Forensic application of this method is based on the comparison of the isotope composition of a target tissue of forensic interest with a local baseline [6–8]. Given that geographic variation in isotope values of drinking water is strongly reflected in δ^18^O values of human tissues [9–11], δ^18^O values of local water are often used as the baseline for interpreting values within tissues. Regardless of the selected analyte for the baseline building (e.g., precipitation, bottled water, tap water), the spatial variability of the isotope values can be predicted and expressed as continuous surface maps or “isoscapes” [12,13], which can then be used to perform probabilistic geographic assignments [14].

A critical factor in isoscape-based geographic assignments is quantification of uncertainty in baseline isoscape values, as this value can vary among locations and influences the strength and specificity of forensic inferences [15,16]. In addition to uncertainty derived from isoscape modeling, sources of drinking water at closely located sites may, in some cases, be derived from multiple, isotopically distinct sources, adding uncertainty related to variation in the δ^18^O values of local drinking water. For example, water isoscapes for multiple sources (groundwater, surface water, and precipitation) have been developed for the contiguous USA (a.k.a., CONUS) and compared, demonstrating that within some regions, δ^18^O values of these sources can differ by many parts per thousand [e.g., 13,17–20]. Moreover, within some regions, drinking water is imported from outside of the local hydrological basin, over distances of hundreds of kilometers, and has δ^18^O values dramatically different from those of locally sourced water [21].

Although the presence and significance of spatiotemporal variation in local tap water isotope values has been recognized [22–25], the prevalence and magnitude of local variation has not been well studied. Where tap water δ^18^O values vary over time, some human tissues (e.g., muscle, tooth enamel) may average these temporal fluctuations, reflecting local means, whereas others with relatively faster turnover rates (e.g., hair, fingernail) may integrate over shorter periods and reflect (e.g., seasonal) variation in δ^18^O values of tap water [9,26,27]. The degree to which human tissues reflect spatial tap water δ^18^O variation within a local area is not well documented, but likely depends on the nature of that variation and the degree to which an individual moves between and ‘samples’ areas of contrasting δ^18^O values.

We hypothesize that isotopic variability within tap water differs across the USA and is systematically correlated with factors that affect the availability and isotopic variability of local water resources. Seasonal variation in precipitation isotope ratios increases with latitude [28] and we hypothesize that this might be reflected in higher tap water δ^18^O interdecile range (IDR, the difference between the 90^th^ and 10^th^ percentile values). Longitude in the contiguous US is strongly correlated with other variables like topographic ruggedness and aridity (see below), and we hypothesize that IDR may decrease from west to east as a result. We hypothesize that developed areas with higher population density and/or median income would have higher IDR because they would have increased demand for and capacity to develop infrastructure to access diverse water resources, respectively. Similarly, we hypothesize that areas with higher per capita water use would have higher IDR. We hypothesize that IDR would decline with increasing access to “local” water resources because communities would be less likely to access diverse, isotopically distinct water sources. Finally, we hypothesized that increasing regional elevation range, or topographic “ruggedness”, would correlate with higher IDR due to elevation-driven variation in the isotopic δ-values of water resources [29,30].

In this study, we use new and previously published stable isotope data from tap water samples collected within 30 discrete developed areas across the contiguous United States and Hawaiian Islands (CONUS-HI). We use the data to explore and quantify spatial and temporal patterns and potential drivers of small-scale drinking water isotope variability. The results highlight systematic relationships with climatic, geographic, and anthropogenic drivers, which may be useful for predicting the magnitude of local variation and its impact in future forensic geolocation work.

## Materials and methods

### Isotope datasets

Samples for this project were collected in early 2020 by a network of researchers and volunteers working in communities across the contiguous USA and Hawai’i. Each sampling group was asked to conduct a synoptic tap water survey of their local “developed area”, consisting of an area of contiguous development ranging from small cities and suburbs to major metropolitan areas. Samples were collected during a single sampling bout spanning a period of days to a few weeks by running a cold water tap at a private residence, business, or public facility for ~15 seconds and then filling a sample vial. Vials were capped tightly and were either analyzed locally or returned to the SIRFER laboratory at the University of Utah for analysis (see below). Collectors compiled standardized metadata documenting each sample, including the location and time of sample collection and the type of water sampled (those samples analyzed here are limited to Type = “Tap”).

Most samples (*n* = 709) were analyzed using a Picarro L2130-i spectroscopic analyzer at the SIRFER laboratory, University of Utah, following the methods described in Good et al. [31]. Briefly, each sample was injected in quadruplicate and data from all four injections were averaged following correction for memory and instrument drift to obtain the uncalibrated sample value. These values were calibrated to the VSMOW-SLAP scale using co-analyzed results from two in-house reference waters, PZ (δ^2^H = +18.1‰, δ^18^O = +1.93‰) and UT2 (-119.1‰, -15.84‰). Long-term reproducibility of measured values for a secondary reference water (EV, δ^2^H = -72.3‰, δ^18^O = -10.16‰) were 0.3‰ for δ^2^H and 0.05‰ for δ^18^O over a ~2-year period spanning the analysis period. Two sets of samples were analyzed by the local laboratory coordinating the sampling (University of Florida, *n* = 30; Vanderbilt University, *n* = 59) using Picarro L2120-i and L2130-i analyzers, respectively. We do not report detailed analytical methods and calibration procedures for these subsets but note that because samples for each community (including those represented in the published work described below) were analyzed at a single lab, and because our data analysis focuses on isotopic variation among samples collected within local communities, minor differences between the methods used at different laboratories should have minimal influence in our study. Although inter-laboratory variability would not affect the main focus of this study (variability across cities in interdecile range), cross-laboratory proficiency testing suggests that stable isotope δ-values for freshwater samples are generally comparable within ~1.5‰ for δ^2^H and 0.2‰ for δ^18^O between laboratories with robust quality control [32].

The new data were combined with additional public datasets documenting spatial and temporal variation across developed areas in the western USA; each of these (Table 1) is available through the Waterisotopes Database (wiDB, https://waterisotopesDB.org). wiDB project 00393 includes tap water data from Los Angeles, Phoenix, Salt Lake City, San Diego, and San Francisco that were used in this study [33]. Published data documenting isotope variation across the Salt Lake Valley urban area [23; wiDB project 00058] were supplemented by two additional years of unpublished data collected by the same authors (project 00059). The samples collected by both Tipple et al. and Jameel et al. were analyzed at the same lab as the new data generated for this study (the SIRFER laboratory, University of Utah). A Kent State University project [34] (wiDB project 00262) focused on water from Ohio; data from Cleveland-Akron and Youngstown were included in this study. Data from these studies were partitioned into sampling bouts that spanned durations similar to those represented in the newly collected data. Data from Salt Lake City represent 11 distinct sampling bouts conducted approximately semiannually between 2013 and 2017; those from Los Angeles represent March-April and November 2014; those from Phoenix represent March-April and October 2014; those from San Diego represent December 2013 and April 2014; Cleveland-Akron was collected in early 2018 (January–March) and mid-year (July–August) and those from San Francisco are derived from seven bouts conducted between 2013 and 2015.

**Table 1:** Datasets used for this research.

Repository ID	Project Name	Reference
00059	Unpublished SLV Tap Water	unpublished
00225	2019 Groundwater	This study
00058	2016 SLV Tap Water Paper	[23]
00393	Isotope and elemental geochemistry of tap waters from several major US metropolitan areas	[33]
00262	NE Ohio tap	[34]

In addition to the isotope measurements and their analytical precisions, all datasets contain geographic and temporal information such as the name of the sampling location, coordinates, elevation, depth, collection date, the facility responsible of the analysis. Deuterium excess was calculated following Dansgaard (29): d-excess = δ^2^H – 8 × δ^18^O.

### Data analysis

Data processing, analysis and visualization were carried out using the R programming environment (version 4.2.1, https://cran.r-project.org/). The data analyzed in this work as well as the R code used to conduct all analyses and generate data figures are available on GitHub [35].

We characterized the magnitude of local isotopic variation within each developed area using the interdecile range (IDR, the difference between the 90^th^ and 10^th^ percentile values). Separately, we characterized the pattern of isotopic variability within each area as either unimodal or multimodal based on the results of a k-means cluster analysis of the local tap water δ^2^H and δ^18^O values using the factoextra R package [36]. We estimated the optimal value of k using the gap statistic; areas for which k = 1 were considered unimodal, and all others multimodal.

Single sampling bouts may not be representative of longer-term patterns within any given developed area. To assess whether estimates of variation and modality derived from single sampling bouts were representative, we examined temporal patterning in two study areas in which numerous repeated sampling bouts were conducted (Salt Lake City and San Francisco). Data from each bout were analyzed separately to determine modality and IDR, and these results were then compared across the bouts to assess their stability over time. For analysis relative to the rest of the dataset we averaged the IDRs and used a majority rule to determine the modality of the tap water δ-values in the six developed areas sampled in multiple bouts (Cleveland-Akron, Los Angeles, Phoenix, Salt Lake City, San Diego, and San Francisco). In the case of a tie, multimodality defined this developed area.

Data for predictors that we hypothesized might be drivers of IDR (latitude, longitude, median income, population, land area, population density, precipitation, streamflow, and ruggedness) were collected from publicly available sources. Values of both latitude and longitude (decimal degrees) were extracted for the midpoint of each developed area. Land area (km^2^) for each developed area was derived from cartographic boundary shapefiles available from the United States Census Bureau. For creating a predictive model of the contiguous US, these areas outside these boundary shapefiles (i.e., outside of Census-recognized metropolitan/micropolitan areas), a base size of 2.5 km was assigned to generalize small rural areas. Population density and median income were extracted within the counties encompassing the sampling locations for each developed area (obtained using the tigris package [37]). We used the censusapi R package [38] to obtain median income and population from the United States Census Bureau’s 2020 5-year American Community Survey. The 5-year survey covers the period 2016–2020 and provides more comprehensive coverage for low-population areas than one-year reports [39]. For those developed areas spanning multiple counties, total population size was calculated as a sum and a mean of median income was calculated across counties, weighting by population. Population density was calculated from total population size divided by the total land area of the associated counties. County-level water use was gathered from a US Geological Survey report and converted to per capita values (L/person/day) [40].

We used local precipitation (mm/year) and streamflow (km^3^/year) as proxies for the abundance of local water resources. Using previously published average annual streamflow [41] and precipitation [42], we calculated the mean of values across all cells of the raster within a 20 km radius and used the mean of these values within the city boundaries of each developed area. Because the streamflow estimates used here were not available outside of the CONUS, we excluded data from Oahu and Hawai’i from our modeling.

We used the elevatr package to obtain digital topographic data at 1.7 km resolution for the CONUS. For each grid cell in the elevation raster, we calculated the range (maximum ‐ minimum) of elevation values across all cells within a 100 km radius. For model fitting, we extracted the maximum roughness value for all grid cells within the city boundaries of each developed area in our study.

We implemented these variables as linear predictors for the square root of IDR of δ^18^O and conducted a model selection process using the regsubsets function in the leaps R package [43]. IDR values were square-root transformed to normalize their distribution and enforce positive values for IDR predictions. Values for population density, water use, and streamflow were strongly positively skewed and were natural log transformed prior to analysis. Briefly, regsubsets conducts a parameter search to determine the combination of predictor variables that leads to the best adjusted R^2^. We used the exhaustive search method and retained the single best model of each size (from 1 to 9 covariates).

## Results

### Temporal patterns of variability

**Fig 1:**
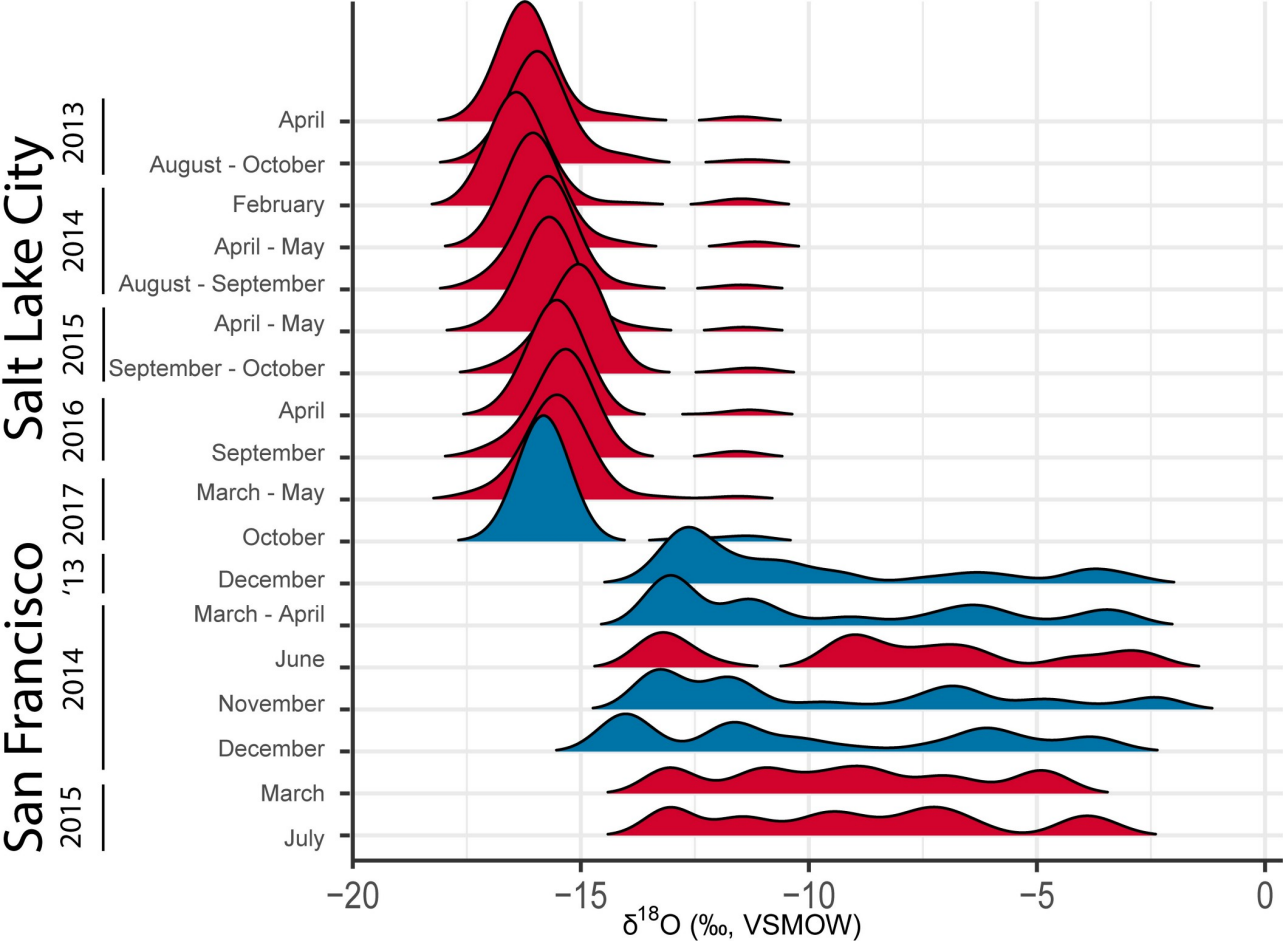
Density plots of δ^18^O from Salt Lake City and San Francisco for different sampling bouts. Multimodal distributions are plotted in blue, unimodal in red.

Interdecile ranges differed consistently between Salt Lake City and San Francisco but were generally similar across time within each city (**Fig 1**), despite major variations in climate over the sampling periods represented at each site [23,25]. Tap water δ^18^O IDRs in Salt Lake City ranged from 0.6–1.7‰, averaging 1.1‰, across the eleven sampling periods. IDR values for San Francisco were consistently much higher, ranging from 8.1–10.2‰ and averaging 9.3‰ across the seven sampling periods. Although the IDR for each area varied somewhat across the sampling bouts, this variation was quite small in comparison with the IDR difference between the two areas. IDR did not exhibit regular seasonal changes in either city, but in both cities did show weak but coherent variation over time (i.e., IDR transitioned between higher and lower values progressively over time). These results suggest that, to first order, results from single sampling bouts can be used to characterize typical levels of tap water isotope δ-value variation within developed areas.

The modality of the tap water δ^18^O distributions in these two areas, in contrast, was variable over time. All Salt Lake City bouts except one were identified as unimodal, and in the San Francisco Bay Area four of seven bouts were classified as multimodal (**Table 2**). Previous in-depth study of water isotope distributions in both areas has clearly demonstrated the presence of multiple, isotopically distinct water sources and their imprint on tap water values across the developed area [23,25,33]. This imprint is visually apparent in the δ-value distributions reported here (**Fig 1**). Regardless, the clustering algorithm used here did not detect the presence of multiple modes in many cases, perhaps due to uneven sampling across the distribution of δ-values or blending between the modes due to mixing of water from multiple sources. This result suggests that our analysis likely provides a conservative estimate of the prevalence of multi-modal tap water isotope distributions, and may fail to identify some cases where multiple, isotopically distinct water sources contribute to isotopic variation within a developed area.

**Table 2:** Interdecile range of δ^18^O, δ^2^H, and d-excess and number of identified clusters for the sampling bouts in Salt Lake City and San Francisco, in temporal order.

Developed Area	Sampling Period	IDR δ^18^O (‰)	IDR δ^2^H (‰)	IDR d-excess	Clusters	*n*
Salt Lake City	Apr 2013	0.8	4.8	5.0	1	153
	Aug–Oct 2013	1.7	5.2	6.3	1	197
	Feb 2014	1.2	6.6	6.7	1	140
	Apr–May 2014	1.0	5.8	6.5	1	216
	Aug–Sept 2014	0.9	4.1	4.5	1	178
	Apr–May 2015	0.9	4.8	5.3	1	202
	Sept–Oct 2015	1.2	5.0	4.8	1	140
	Apr 2016	1.0	5.0	5.0	1	128
	Sept 2016	1.0	6.4	5.1	1	139
	Mar–May 2017	1.3	5.4	5.6	1	177
	Oct 2017	0.6	2.9	3.1	2	81
San Francisco	Dec 2013	8.9	70.4	4.6	3	60
	Mar–Apr 2014	9.4	72.0	5.6	5	204
	June 2014	10.2	78.2	6.9	1	86
	Nov 2014	9.7	73.4	7.8	2	101
	Dec 2014	9.8	74.0	7.3	2	97
	Mar 2015	8.1	65.0	5.6	1	90
	July 2015	9.0	71.3	6.9	1	88

### Isotopic and spatial patterns of variability

**Fig 2:**
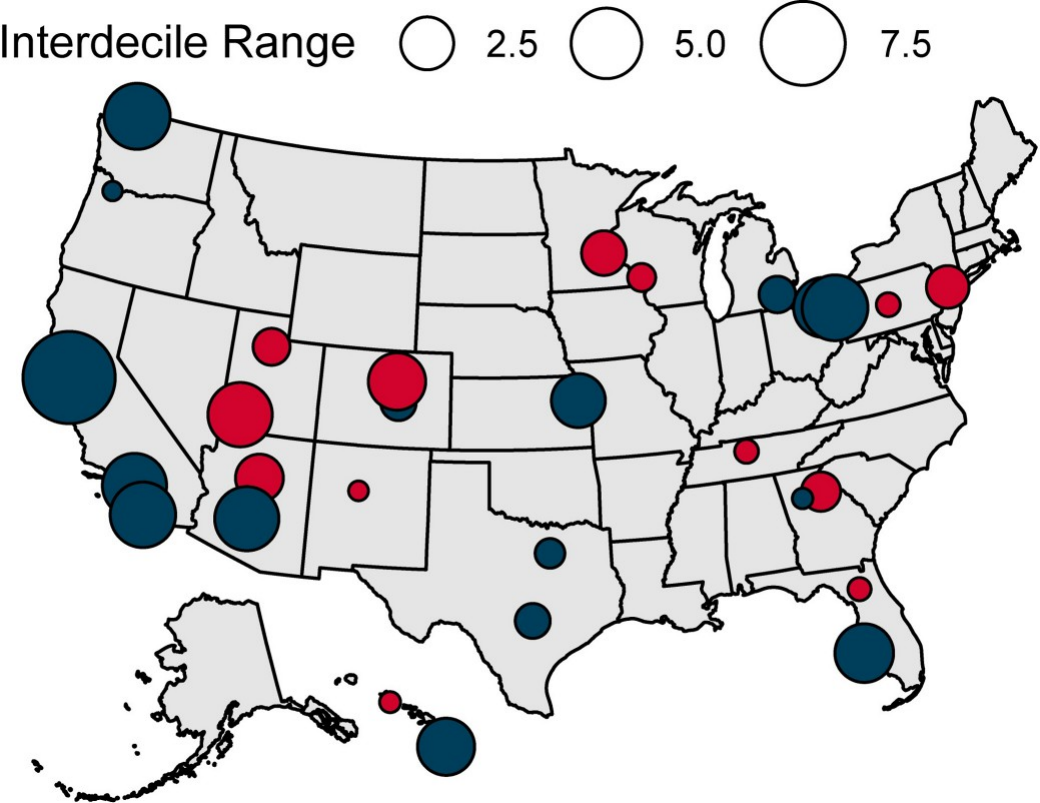
Location, magnitude of δ^18^O IDR, and type of variability from the sampling sites. Red sites are unimodal and blue sites are multimodal.

The full dataset comprises 4,068 measurements of samples from 30 urban areas throughout CONUS-HI (**Table 3)**. The largest interdecile ranges (4.0–9.3 δ^18^O IDR) were observed in the western part of the contiguous United States (**Fig 2**; i.e., Cedar City, Los Angeles, Phoenix, San Diego, Bellingham, San Francisco). Smaller ranges were generally observed across the southern and much of the eastern CONUS, although moderately high IDR values were observed interspersed throughout these regions. There was no relationship between number of samples within a given developed area and IDR (*r*(26) = 1.52, *p* = 0.141). Modality and IDR were not strongly correlated and exhibited different spatial patterns: the 16 unimodal developed areas are mostly scattered around the Midwest and East Coast, whereas multimodal tap water distributions (*n* = 14) are more common across the southern Great Plains and the West Coast.

**Table 3:** Median and interdecile range of δ^18^O, δ^2^H, and d-excess for the studied urban areas, as well as n observations.

		δ^18^O		δ^2^H		Deuterium Excess		N	Modality
Studied Urban Areas	State	Median	IDR	Median	IDR	Median	IDR		
Bellingham	Washington	-8.67	4.3	-66.01	10.7	-8.67	23.5	25	Multi
Portland	Oregon	-9.67	0.2	-65.33	1.6	-9.67	1	43	Multi
Minneapolis	Minnesota	-8.38	1.6	-60.36	7.9	-8.38	5.1	33	Uni
La Crosse	Wisconsin	-8.71	0.5	-58.8	2.1	-8.71	1.6	30	Uni
Ann Arbor	Michigan	-8.24	1	-56.49	1.8	-8.24	5.4	12	Multi
Cleveland-Akron	Ohio	-6.84	2.7	-51.44	11.1	-6.84	11.4	172	Uni
Youngstown	Ohio	-5.81	3.1	-41.83	17.1	-5.81	8.6	80	Uni
Wooster	Ohio	-7.83	0.1	-50.61	0.9	-7.83	0.7	26	Uni
State College	Pennsylvania	-9	0.3	-58.17	2.1	-9	0.7	29	Uni
Morristown	New Jersey	-6.37	1.5	-41.99	4.3	-6.37	7.6	32	Uni
Salt Lake City	Utah	-15.76	1.6	-119.14	8.1	-15.76	6	1752	Uni
Denver	Colorado	-15.16	3	-116.69	17.7	-15.16	5.8	41	Uni
Lawrence	Kansas	-4.4	2.6	-28	19.9	-4.4	1.8	29	Multi
Colorado Springs	Colorado	-15.9	0.9	-120.56	5.9	-15.9	0.8	24	Multi
San Francisco	California	-9.84	9.2	-74.58	70.8	-9.84	6.8	726	Multi
Cedar City	Utah	-14.09	4	-103.14	12.7	-14.09	19.6	30	Uni
Nashville	Tennessee	-5.8	0.3	-34.8	1.1	-5.8	1.6	56	Uni
Flagstaff	Arizona	-10.56	1.9	-79.29	8.7	-10.56	6.6	27	Uni
Albuquerque	New Mexico	-12.5	0.2	-93.28	1.1	-12.5	0.7	32	Uni
Los Angeles	California	-8.9	4.3	-62.73	47	-8.9	15.5	239	Multi
Athens	Georgia	-3.97	1.2	-22.15	6.4	-3.97	2.9	26	Uni
Atlanta	Georgia	-4.92	0.2	-26.44	1.2	-4.92	0.6	48	Multi
Phoenix	Arizona	-9.47	4.2	-71.24	35.5	-9.47	10.7	324	Multi
San Diego	California	-11.43	4.3	-92.74	35.3	-11.43	5.1	91	Multi
Dallas Fort Worth	Texas	-1.51	0.6	-9.72	2.5	-1.51	2.5	17	Multi
San Marcos	Texas	-3.44	0.9	-20.58	4.2	-3.44	3	28	Multi
Gainesville	Florida	-2.09	0.3	-8.82	1	-2.09	2.8	30	Uni
St Petersburg	Florida	-1.27	3.2	-6.1	7.9	-1.27	18	20	Multi
Oahu	Hawai’i	-2.96	0.2	-10.48	2.4	-2.96	2.1	34	Uni
Hawai’i	Hawai’i	-4.47	3	-21.13	26.7	-4.47	6.8	12	Multi

Urban areas ordered from north to south. Note some urban areas cross state lines, but we list the main state.

**Fig 3:**
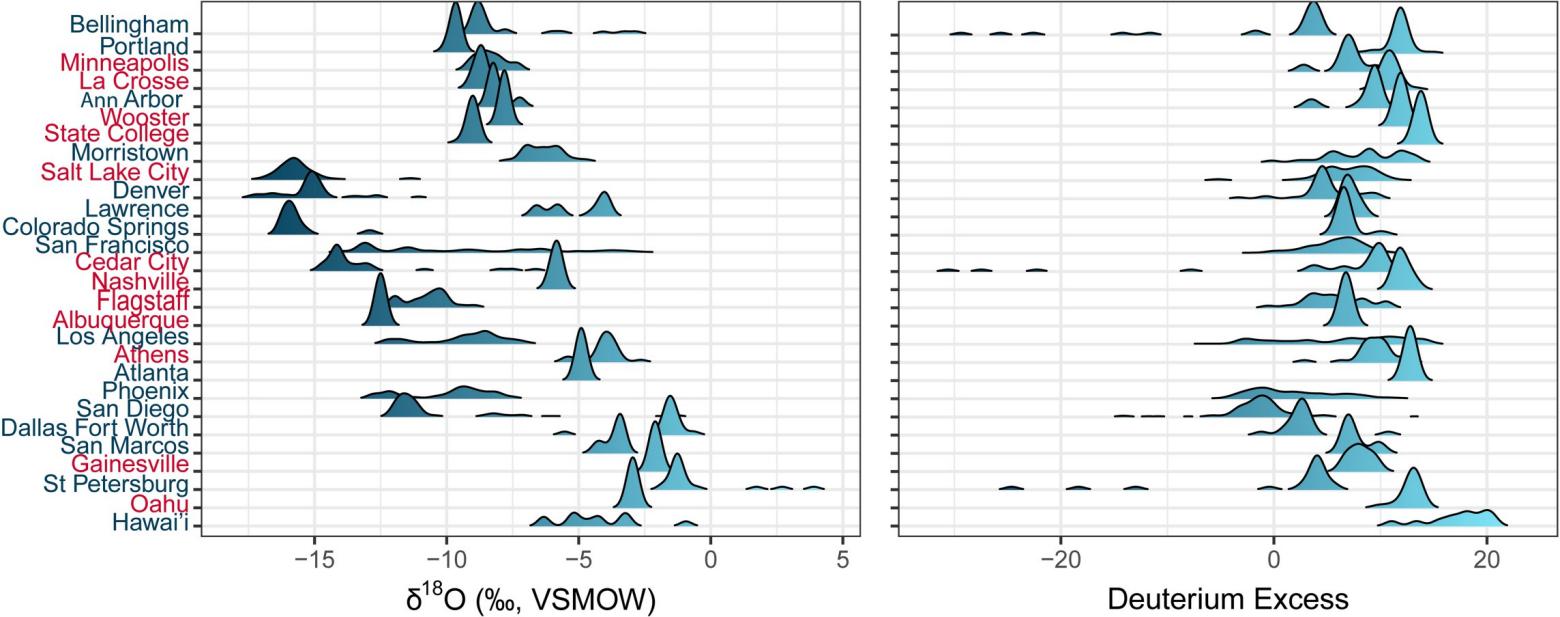
Kernel density estimates of δ^18^O and d-excess from the tap water data, with developed areas organized from north to south. The colors of the developed area names refer to the type of variability (red: Unimodal, blue: Multimodal).

The observed isotope distributions show several patterns across the sampling network, which likely reflect regional hydrological controls on urban tap water isotope values (**Fig 3**). First, δ^18^O (and δ^2^H, not shown) values generally decrease with increasing latitude. This pattern has been demonstrated previously [44] and can be attributed to large-scale latitude-δ^18^O relationships in meteoric precipitation. Second, tap water δ^18^O values of developed areas in and around mountainous areas in the western CONUS are systematically offset from those at similar latitudes elsewhere in the country. This likely reflects the importance of water sourced from high-elevation mountain precipitation in these areas (also previously demonstrated by Bowen et al., 2007). Third, within a subset of the sampled areas δ^18^O distributions are positively skewed and include a thin tail of values substantially higher than the mode. In most cases, these same developed areas also have negatively skewed d-excess distributions. The combination of low d-excess and high δ^18^O values is consistent with evaporative fractionation of these samples and suggests that a subset of tap waters sampled in these areas may be derived from reservoirs or other sources that experienced substantial evaporation [45,46]. Within many of these developed areas the use of both evaporated and unevaporated water sources substantially increases variation in tap water δ^18^O values.

### Environmental and demographic correlations of variability

**Fig 4:**
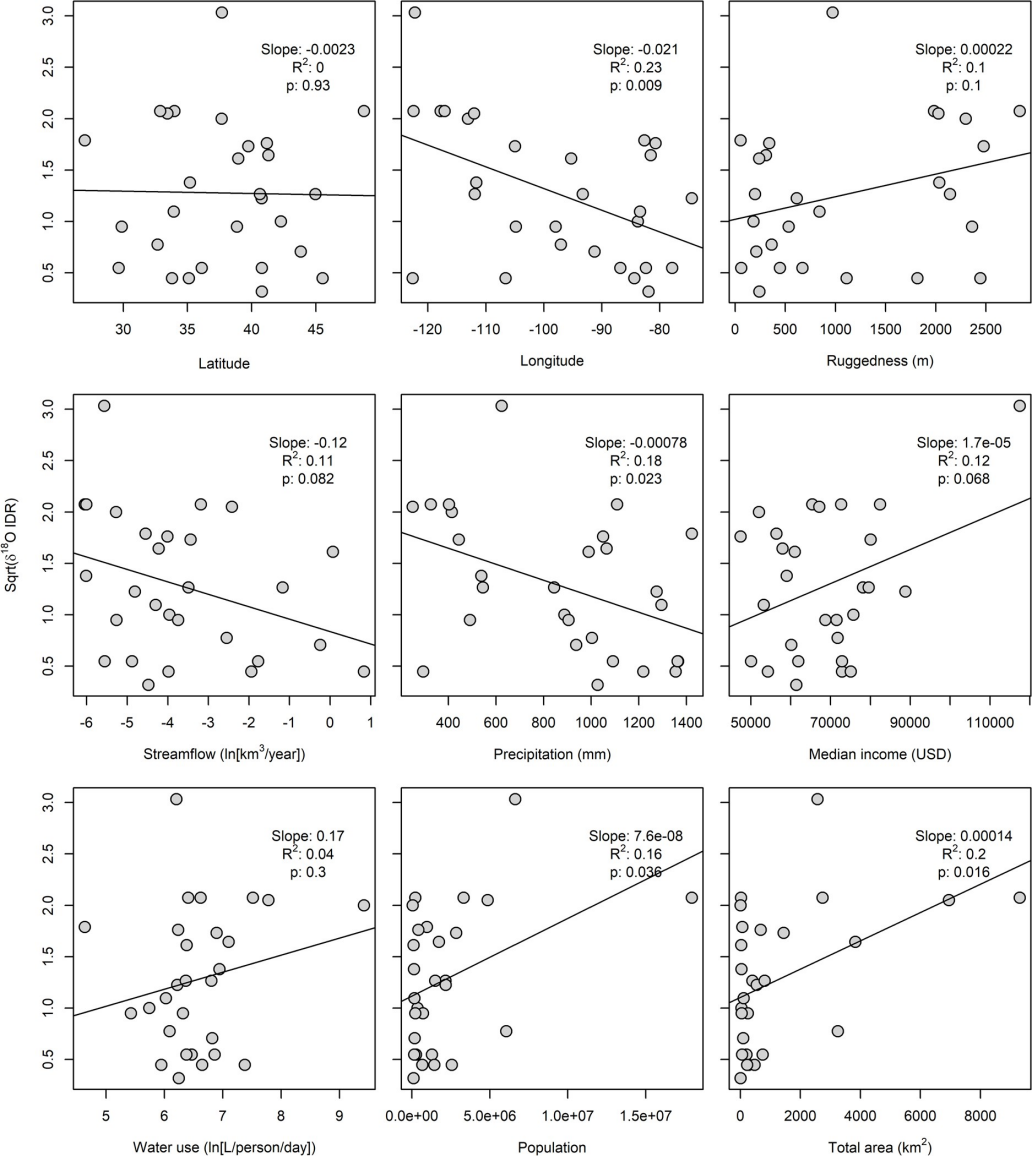
Univariate correlation between potential predictor variables and δ^18^O interdecile range for tap water in CONUS developed areas.

Four of the nine predictors tested here (longitude, *p* = 0.009; precipitation, *p* = 0.023; population, *p* = 0.036; and total area, *p* = 0.016) were significantly correlated with δ^18^O IDR^-2^ at the 95% confidence level (**Fig 4**). The strongest predictor (longitude) explained 23% of the variance in the dataset. In each case, the nature of the observed correlation is consistent with the hypothesized relationship between the variable and IDR: higher IDR^-2^ values are observed in the western CONUS and in developed areas with higher population and area, while there is a negative relationship between IDR^-2^ and precipitation. Several other variables exhibited weak relationships with δ^18^O IDR^-2^ that were significant at the 90% confidence level and consistent in form with our hypothesized mechanisms: higher IDR^-2^ values were observed in developed areas with lower streamflow and higher median income (**Fig 4**). No significant or marginal association was observed between IDR^-2^ and latitude, ruggedness, or water use.

**Fig 5:**
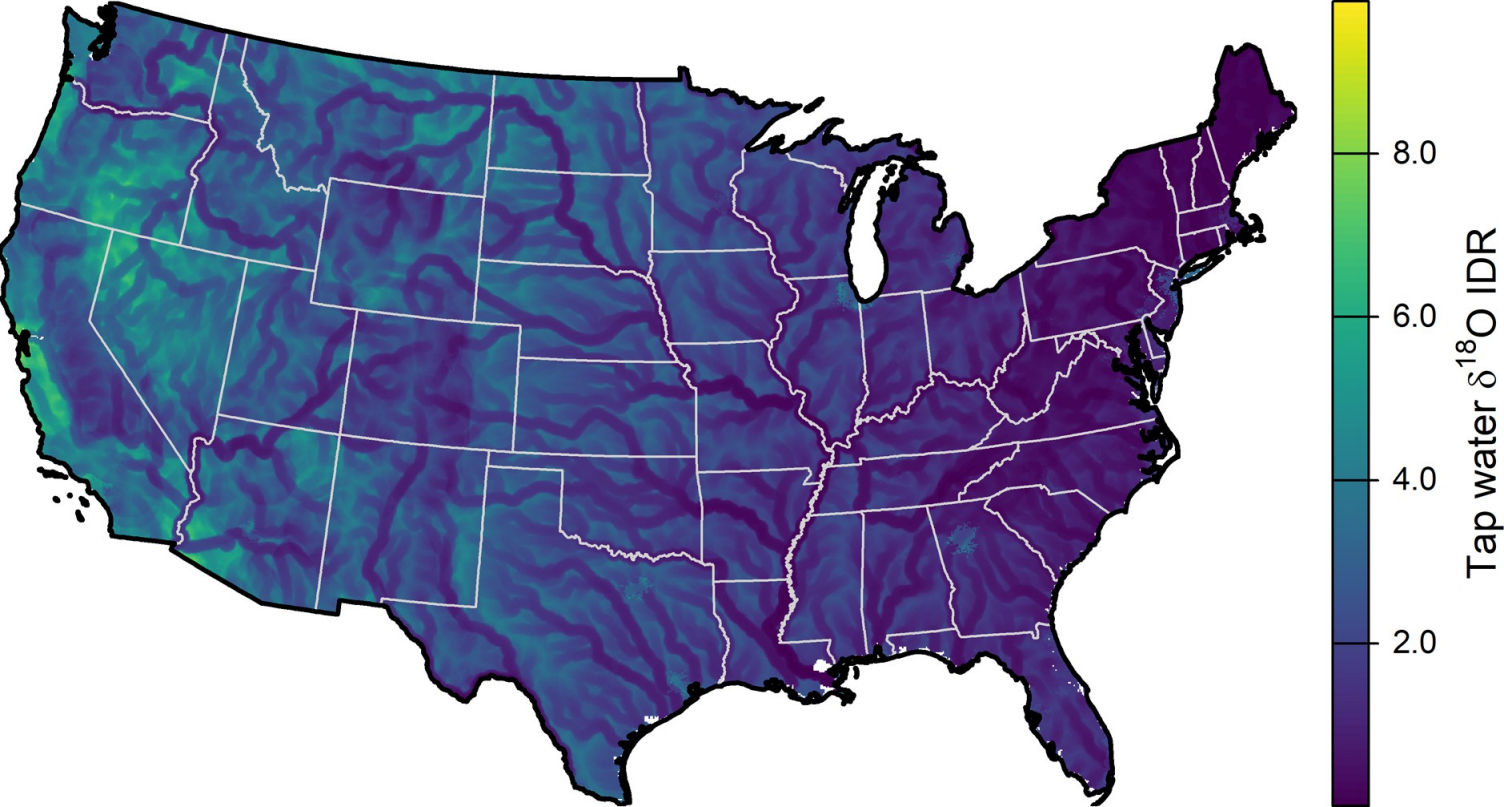
Predictive model of δ^18^O IDR across the contiguous United States, based on longitude, ruggedness, streamflow, and total area.

When all nine predictors were included, the model explains 56% of the observed variance in δ^18^O IDR^-2^ with an adjusted R^2^ = 0.34 (*p* = 0.044). This model is highly over-parameterized and multicollinear, however, as reflected by variable inflation factor values exceeding 6. We selected an optimal model using the regsubsets R package (Table 4). This model was a compromise solution that featured the second-highest adjusted R^2^ (0.42) and second lowest Bayesian information criterion score (in each case the statistic was only marginally different from the highest or lowest score). The model included longitude (negative relationship with IDR^-2^), ruggedness (positive), streamflow (negative), and total area (positive) as predictors. The model had a *p*-value of 0.0002, explained 52% of the observed IDR^-2^ variance, and had low variable inflation factors (< 1.15). Model residuals were normally distributed (Shapiro-Wilk’s test, W = 0.95, *p* = 0.16). We used the optimal model to predict IDR values for tap water δ^18^O across the contiguous USA (**Fig 5**).

**Table 4:** Model selection results, showing the included covariates, Bayesian information criterion (BIC), and adjusted R^2^ (adjR^2^) scores for optimal models retaining from 1 to 9 covariates.

#	Lat	Lon	Rug	Sflow	Precip	Inc	Wuse	Pop	Area	BIC	adjR^2^
1		x								-0.83	0.21
2		x		x						-2.46	0.31
3		x	x	x						-2.80	0.37
4		x	x	x					x	-3.03	0.42
5	x	x	x	x					x	-1.23	0.42
6	x	x	x	x				x	x	0.87	0.42
7	x	x	x	x		x		x	x	3.69	0.41
8	x	x	x	x	x	x		x	x	7.01	0.37
9	x	x	x	x	x	x	x	x	x	10.33	0.34

Lat = latitude, Lon = longitude, Rug = ruggedness, Sflow = streamflow, Precip = precipitation, Inc = median income, Wuse = water use, Pop = population, Area = total area.

## Discussion

### Patterns and drivers of within-city water isotope variation

**Fig 6:**
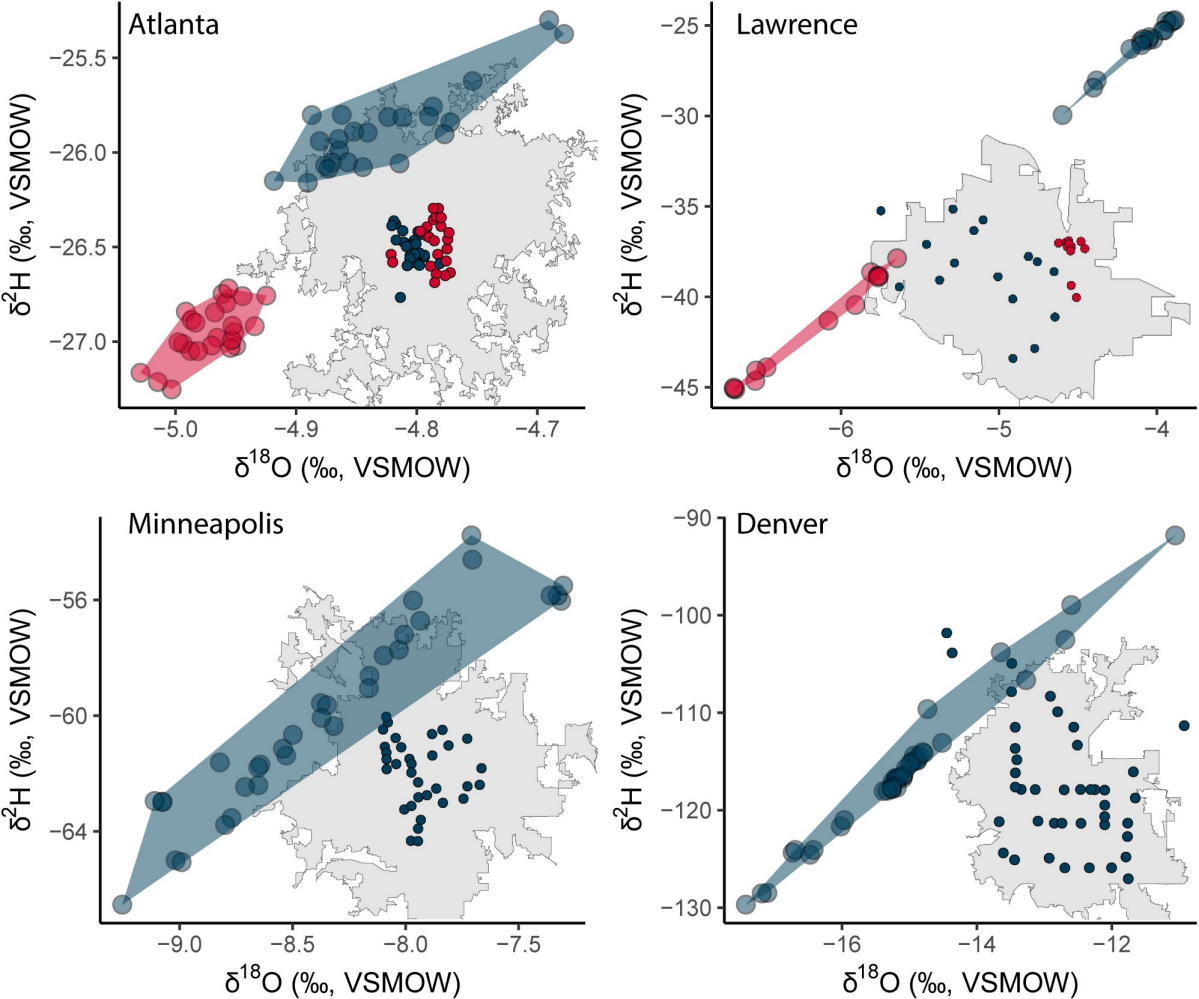
Example δ^18^O and δ^2^H variation in four vignette cities illustrating different range and modality characteristics: Small-range multimodal (Atlanta, GA), large-range multimodal (Lawrence, KS), small-range unimodal (Minneapolis, MN), and large-range unimodal (Denver, CO). The inset in each plot shows sample sites, colored by cluster, superimposed on the municipality boundaries. Note that the range of values displayed on the axes differs in each plot.

We illustrate characteristic patterns of tap water isotope variability within four “vignette cities” that represent different combinations of high-/low-IDR and uni-/multi-modal distribution (**Fig 6**): Atlanta (Georgia), Lawrence (Kansas), Minneapolis (Minnesota), and Denver (Colorado). Atlantaand Lawrence were both classified as multimodal in our analysis, but Lawrence displays a much wider range of δ-values (Atlanta δ^18^O IDR = 0.2‰, Lawrence IDR = 2.6‰). The multimodal nature of Lawrence tap water δ^18^O values is likely a result of the two water sources used in this area: the Kaw River Water Treatment Plant draws from the Kansas River, whereas the Clinton Reservoir Water Treatment Plant sources from the Clinton Reservoir on the Wakarusa River [47]. The contrasting size and different drainage basins of these river, potentially along with differences in upstream water management, likely give rise to the different δ^18^O values characterizing these two sources. Atlanta is served by three major water treatment plants, and all use water that is ultimately derived from the Chattahoochee River. The general distribution of samples associated with the more evaporated (lower d-excess) mode in Atlanta matches descriptions of the service region for the Hemphill Water Treatment Plant, which draws its water from intermediate reservoir storage, in contrast to the other two plants which draw directly from the river [48]. We suggest that the common source, coupled with a small amount of evaporative loss during reservoir storage, explains the combination of the small range of values and multimodality observed in this area. In both cases, tap water samples assigned to the different δ-value modes are spatially clustered, consistent with water from different sources being distributed to different regions within the developed area as previously shown in several western U.S. cities [23–25,33].

Our two unimodal model cities are Denver and Minneapolis, with Denver displaying wider range of values (δ^18^O IDR = 3.1‰) and Minneapolis having a more restricted range of values (δ^18^O IDR = 1.7‰). Denver’s supply system relies on water sourced from approximately 6,500 km^2^ of the Rocky Mountain range west of the urban area and stored in 12 major reservoirs [49], creating substantial potential for isotopic variation between sources that has previously been shown to contribute to tap water isotopic variation across this developed area [24]. The absence of clear multi-modal clustering within the Denver dataset may reflect a combination of mixing between sources during water treatment and distribution and the details of our sample collection, which returned many samples with similar isotopic composition and a distributed scatter of samples with higher or lower values. Minneapolis, in contrast, largely draws its water from the Mississippi River and three aquifers proximal to the city and river [50]. Although multiple sources are used, the relatively low range of tap water δ^18^O values and lack of multimodality likely reflects the limited isotopic variation among these sources.

Our results show that multimodal tap water isotopic variation within individual developed areas derives primarily from the widespread use of multiple, isotopically distinct water sources and is common across the CONUS-HI. In combination with visual evidence showing that our k-means-based method was conservative in detecting multimodal distributions, this suggests that a large fraction of USA developed areas likely exhibit measurable tap water isotopic variation. This is true even in cases where the differences in the history and isotopic composition of these waters are minor, as in the Atlanta case study. This implies potential for the widespread use of water isotopes as tracers in urban hydrology and environmental forensics, in that that propagation of water from different sources through distribution systems and with the urban environment may be traceable using H and/or O isotopes [e.g., 24,51].

The observed range of δ^18^O variation was dramatically different across the 30 developed areas studied here. Repeated sampling within two cities showed that IDR values within a given urban area are not identical over time, but that temporal variation in IDRs was small relative to the range of IDRs measured across our sampling network. This implies that tap water isotopic variation may be a characteristic of different cities and towns that emerges from a combination of environmental and structural properties of the water supply system and is conserved over time.

Tap water δ^18^O IDR values were somewhat higher, on average, in the western USA, where there is substantial topographically-driven variation in rainwater isotope values that is known to be reflected in tap water isotope values (Bowen et al., 2007). Indeed, longitude and topographic roughness were two of the strongest predictors in the multivariate model for IDR, supporting the importance of topography as a driver of tap water isotope variation. Developed areas with relatively low IDR also occur across the western USA, however, and several cities with quite high tap water isotope IDR are found in the eastern states. For example, Youngstown (Ohio) has both reservoir and groundwater sources and a high IDR (δ^18^O IDR = 3.1‰), while ~100 km east Wooster has a very small IDR (δ^18^O IDR = 0.1‰). Thus, the evidence suggests a more complex and multi-faceted set of factors govern the range of isotopic values seen within local tap water systems.

Our analysis suggests two additional types of controls that that may modulate the large-scale pattern of tap water isotope variation across the CONUS. First, climatic and hydrological factors that affect the availability of local water resources may influence whether communities invest in developing infrastructure to access multiple, isotopically distinct water sources. Tap water δ^18^O IDR was lower in developed areas with higher local precipitation and streamflow (although streamflow’s univariate relationship was only significant at the 90% confidence level), and streamflow was retained as a predictor in our optimal multivariate model. Although water supply diversification may have stability and resilience benefits, as demonstrated, for example, in the management of multiple reservoirs to reduce climate and earthquake risk exposure in the San Francisco Bay Area [25], its development can be costly, particularly where infrastructure accessing remote resources (e.g., deep aquifers, inter-basin transfer) is required [52]. Our result suggests that such diversification may be less likely in developed areas where local (and thus isotopically similar) water sources are abundant. Second, socioeconomic factors may also be reflected in the tendency of developed areas to build infrastructure to access diverse water resources. We found that tap water δ^18^O IDR was positively correlated with total population, total land area, and median household income (the latter at the 90% confidence level). These relationships suggest that the growth of communities, whether in terms of size, population, or affluence, may be associated with the use of more (isotopically) diverse water resources. Possible mechanisms include growth of demand, assimilation of new local water resources as an urban area’s footprint grows, and investment in infrastructure development projects as a solution to local water resource scarcity.

### Predictive model and implications for forensic geolocation

We were able to develop a relatively simple, multivariate linear model that explained approximately 50% of the observed variation in tap water δ^18^O IDR across the CONUS. Only four variables (longitude, ruggedness, streamflow, and total area) were retained in the optimal model, and collectively these reflect the three primary classes of controls on tap water isotope variability discussed above (regional isotope heterogeneity, availability of local water resources, and demographic factors, respectively). Predictions from the model range from IDR values close to 0‰ to value slightly greater than 10‰ (**Fig 5**), dominated by a broad east-to-west increase. Across most of the country, communities situated near major surface water sources have low predicted IDR values (< 2‰). More populous urban areas tend to stand out within their regional context, particularly within the western USA (e.g., Denver and the San Francisco Bay area). Although the model exhibits a reasonably strong fit to the available data, we acknowledge that several major CONUS metropolitan areas (e.g., the Northeast megaregion, metro Chicago and Houston) were not sampled here; in these cases the model provide a prediction that could be tested with additional data collection.

The tap water isotope variability documented here represents one source of potential complexity for forensic geolocation applications in that this variability could lead to isotopic heterogeneity in the bodily tissues of different individuals living within these local areas. If unrecognized, this local heterogeneity could lead to misinterpretation of data in forensic casework: for example, the exclusion of a particular location as possible origin for an individual whose body tissue values do not match mean predictions for that location because they are influenced by a non-local water source. Understanding of the potential and limitations of oxygen isotopes in human identification is still being developed, but data on natural variability and analytical uncertainty for different body tissues suggests that, currently, differences in δ^18^O values of less than 1 or 2‰ are too small to support meaningful interpretations [9,53]. Our predictive model suggests that tap water δ^18^O IDR is less than 1‰ across 20% of the CONUS land area, and less than 2‰ over 52% of the CONUS land area. This implies that if tap water variability is transferred directly into isotopic heterogeneity among local residents this phenomenon would have substantially different impacts on the uncertainty and potential error of isotope-based forensic interpretations in different parts of the USA.

Determining whether local variability in tap water isotope δ-values in the remaining, high-IDR, regions of the country is problematic for forensic applications will require further research assessing the degree to which this variability is reflected within local populations. Drinking water is only one source of O and H to the human body (along with O and H derived from food and atmospheric O_2_) [9,54]), and contributions from these other sources would be expected to dilute variation imparted from local tap water. Moreover, drinking water isotope intake by an individual might be homogenized, to some degree, as they move within their local environment, reducing the isotopic contrast between body tissues of individuals living within different parts of a developed area (e.g., as previously suggested for local populations of songbirds [55]. Previous work with strontium isotopes, however, shows that variation in tap water isotope values within a single urban area can be transferred to human hair [26], suggesting that the local tap water H and O isotope variability studied here may be of relevance to interpretations of forensic data in some settings. Given this, we suggest that our predictive map (Fig 5) can be used as a screening tool to conservatively identify areas where substantial tap water isotope variation is likely to exist and may need to be considered in the interpretation of forensic data. As future work refines our understanding of the transfer of local (e.g., within-city) H and O isotope variation into body tissues, this information can be added to the model presented here to develop refined predictions of the expected isotopic variation in local populations and more precise geolocation interpretations.

## Conclusion

Our work shows that the magnitude of spatial variation in tap water H and O isotope values within urban areas ranges dramatically across the USA. This variation is largely attributable to the use of multiple, isotopically distinct water sources, and its magnitude is relatively (though not completely) stable over time within a given developed area. Even in cases where the amount of variability is small, multiple isotopic modes can often be detected, suggesting that use of multiple water sources is a prevalent feature in U.S. cities and that, to a large degree, the magnitude of isotopic variability may be controlled by the isotopic heterogeneity of available water sources. This is supported by our modeling results, which highlight correlations between O isotope variation within developed areas and geographic variables associated with isotopic heterogeneity in water resources. Predictive modeling of δ^18^O variability across the CONUS suggests that the magnitude of local variation is relatively small in most locations but could be large enough to bias forensic interpretations across ~50% of the country (primarily within the western USA). Given this, we encourage conservative forensic interpretation of H and O isotope data from these regions and suggest further research to clarify understanding of the degree to which local heterogeneity in tap water isotope δ-values is reflected in human tissues.
